# The Role of Cognition, Affective Symptoms, and Apathy in Treatment Adherence with Noninvasive Home Mechanical Ventilation in Myotonic Dystrophy

**DOI:** 10.3233/JND-240081

**Published:** 2024-09-03

**Authors:** Bettine A.H. Vosse, Jasmijn de Jong, Leandre A. la Fontaine, Corinne G.C. Horlings, Sander M.J. van Kuijk, Nicolle A.M. Cobben, Anne-Fleur Domensino, Caroline van Heugten, Catharina G. Faber

**Affiliations:** a Department of Pulmonary Diseases and Home Mechanical Ventilation, Maastricht University Medical Centre+, Maastricht, The Netherlands; b Department of Neurology, Maastricht University Medical Centre+, Maastricht, The Netherlands; c Department of Neurology, Medical University Innsbruck, Innsbruck, Austria; d Department of Clinical Epidemiology and Medical Technology Assessment, Maastricht University Medical Centre+, Maastricht, The Netherlands; e School for Mental Health and Neuroscience (MHeNS), Maastricht University, Maastricht, The Netherlands; f Limburg Brain Injury Centre, Maastricht, The Netherlands; g Department of Neuropsychology and Psychopharmacology, Faculty of Psychology and Neuroscience, Maastricht University, Maastricht, The Netherlands

**Keywords:** Treatment adherence, myotonic dystrophy, respiratory insufficiency, artificial respiration, cognitive dysfunction, apathy, affective symptoms

## Abstract

**Background:**

Chronic respiratory failure often occurs in myotonic dystrophy type 1 (DM1) and can be treated with noninvasive home mechanical ventilation (HMV). Treatment adherence with HMV is often suboptimal in patients with DM1, but the reasons for that are not well understood.

**Objective:**

The aim of this exploratory study was to gain insight in the prevalence of mild cognitive impairment, affective symptoms, and apathy and to investigate their role in HMV treatment adherence in DM1.

**Methods:**

The Montreal Cognitive Assessment (MoCA), the Hospital Anxiety and Depression Scale (HADS), and the Apathy Evaluation Scale (AES) were used to assess cognition, affective symptoms, and apathy in DM1 patients that use HMV. Patients with low treatment adherence (average daily use HMV <5 h or <80% of the days) were compared with patients with high treatment adherence (average daily use of HMV≥5 h and ≥80% of the days).

**Results:**

Sixty patients were included. Abnormal scores were found in 40% of the total group for the MoCA, in 72–77% for the AES, and in 18% for HADS depression. There was no difference between the high treatment adherence group (*n* = 39) and the low treatment adherence group (*n* = 21) for the MoCA, AES, and HADS depression. The HADS anxiety was abnormal in 30% of the total group, and was significantly higher in the low treatment adherence group (*p* = 0.012). Logistic regression analysis revealed that a higher age and a higher BMI were associated with a greater chance of high treatment adherence.

**Conclusions:**

This exploratory study showed that cognitive impairment and apathy are frequently present in DM1 patients that use HMV, but they are not associated with treatment adherence. Feelings of anxiety were associated with low treatment adherence. Higher age and higher BMI were associated with high treatment adherence with HMV.

## INTRODUCTION

Myotonic dystrophy type 1 (DM1) is the most common muscular dystrophy among adults. It is a hereditary autosomal dominant disorder affecting not only the peripheral muscles, but also multiple organ systems including the respiratory system, the heart, and the central nervous system [1, 2]. Chronic respiratory failure (CRF) often occurs and can be treated with noninvasive home mechanical ventilation (HMV) [3]. In DM1, HMV improves gas exchange and relieves symptoms with a possible survival benefit [4, 5]. HMV can, however, only be applied successfully to cooperative and motivated patients, and its effect depends on the amount of hours the ventilator is used every night, which is referred to as treatment adherence [5]. Unfortunately, treatment adherence with HMV is often suboptimal in DM1 patients. Low treatment adherence (defined as use of the ventilator less than 5 hours per night) and discontinuation of HMV is reported in 24–72% of DM1 patients [6–12]. The reasons for low treatment adherence with HMV in DM1 are not well understood, but they are not solely determined by physiological factors or the severity of the respiratory dysfunction [9]. Patients who report symptomatic benefit have higher treatment adherence than those who do not feel benefit [12]. Identifying additional influencing factors can potentially result in new modifiable targets to enhance treatment adherence. There is currently very limited data available on the role of cognition, affective symptoms, and apathy in the context of HMV treatment adherence [13]. Cognitive impairment, depressive and anxiety symptoms, and apathy are known to be major predictors of poor treatment adherence, and they are often present in DM1 patients [14–19]. Also, lack of treatment adherence cannot be fully understood without taking into account the social context in which it occurs, including the living situation and care dependency of the treated patient [20, 21]. The aim of this exploratory study was to gain insight in the prevalence of mild cognitive impairment, affective symptoms, and apathy and to investigate their role in HMV treatment adherence in DM1. We hypothesized that the presence of cognitive impairment, affective symptoms, and apathy is common in DM1 patients, and that it is negatively associated with treatment adherence with HMV.

## MATERIALS AND METHODS

### Study design and participants

A cross-sectional study was conducted at the Home Mechanical Ventilation (HMV) center of the Maastricht University Medical Center+ (MUMC+). Data was collected between October 2021 and October 2022 during a regular follow-up visit in the hospital or during a regular home consultation. The local Medical Ethics Committee of the MUMC+concluded that the study protocol falls outside the scope of the Medical Research Involving Human Subjects Act (registration number METC 2021–2665). The study was conducted according to the applicable research principles. Written informed consent was obtained from all included participants. All DM1 patients who were treated with HMV at the HMV Center of MUMC+were approached to take part in the study. HMV initiation and treatment were in accordance with the current consensus-based care recommendations for DM1 patients [22]. To avoid selection bias, patients who had recently (within the past year) discontinued HMV were also asked to participate. Patients were required to meet the following criteria to be eligible for participation: (1) having confirmed DM1, (2) undergoing HMV treatment for at least three months, or having undergone HMV treatment, but discontinued in the past year, (3) ≥18 years old, (4) no recent history of pulmonary infection or hospitalization, (5) able to read and write the Dutch language, and (6) able to give informed consent.

### Materials

Patient characteristics such as age, sex, body mass index (BMI), CTG (cytosine-thymine-guanine) repeat length, pulmonary function (forced vital capacity sitting and supine), pCO_2_, and apnea-hypopnea-index (AHI) were obtained from the patients’ medical file. The muscular impairment rating scale (MIRS), a DM1-specific scale for muscle weakness, was used to rate muscular impairment. Ventilator settings and treatment adherence data were retrieved from the patients’ ventilator and expressed as amount of days that the ventilator was used (as percentage of total amount of days that the ventilator could possibly be used), and average time (in minutes) that the ventilator was used per night. Data on the living situation, care dependency, and level of education were collected. Low level of education was defined as primary school, lower vocational education, or secondary vocational education as highest level of education.

### Test battery

An overview of the test battery can be found in Table 1. The Montreal Cognitive Assessment (MoCA) was used as a screening tool for global cognitive functioning. The MoCA is a one-page-30-point test which evaluates eight different domains of cognition: short-term memory, visuospatial abilities, executive functions, attention, concentration, working memory, language, and orientation to time and place. A score below 26 is considered abnormal [23, 24]. The MoCA was carried out by trained researchers. The Hospital Anxiety and Depression Scale (HADS) was used to assess emotional functioning. The HADS is a fourteen-item scale and consists of an anxiety subscale and a depression subscale, each with seven items. The items are rated on a four-point scale ranging from zero to three. A higher score on the scale indicates more (severe) symptoms, and a domain score of seven or more indicates the possible presence of anxiety or depression [25, 26]. To evaluate the presence of apathy, the short version of the Apathy Evaluation Scale (AES) was used. The AES-S is the self-rated version and the AES-I is the informant version based on observations of a family member, friend, or caregiver. The AES contains 14 items that address the affective, behavioral, and cognitive domains of apathy. The items are rated on a four-point scale that ranges from zero (‘not at all present’) to three (‘a lot’). A score of 14 or higher indicates the presence of apathy, and a higher score indicates more symptoms of apathy [27–29]. The Care Dependency Scale (CDS) was used to determine the extent to which a patient is dependent on others for everyday tasks and self-care with a total score ranging from 15 (totally dependent) to 75 (totally independent) [30]. The S^3^-NIV questionnaire was used to determine HMV-related side effects, symptoms and sleep quality. The S^3^-NIV questionnaire is a self-reported questionnaire containing 11 items. Patients score each item on a five-point Likert-scale (zero: always true; one: mostly true; two: sometimes true; three: mostly untrue; four: completely untrue) based on their agreement with each statement in the four preceding weeks. The total score can be computed as the average of all answered items multiplied by 2.5. The lowest possible score (0) corresponds to the highest impact of disease and treatment, while the highest possible score [10] corresponds to the lowest impact of disease and treatment [31, 32].

**Table 1:** Test battery

Test /questionnaire	Measurement
Montreal Cognitive Assessment (MoCA)	Cognitive functioning
Hospital Anxiety and Depression Scale (HADS)	Affective symptoms
Apathy Evaluation Scale (AES)	Degree of apathy
Care Dependency Scale (CDS)	Degree of care dependency
S^3^-NIV questionnaire	HMV related symptoms, side effects, and sleep quality

### Statistical analysis

Statistical analysis was performed using IBM SPSS statistics software version 28. Continuous variables were expressed as mean with standard deviation (SD) or as median with 1st and 3rd quartile in case of skewed distribution. Categorical variables were expressed as count and percentage. Patients were stratified into two group based on their treatment adherence. The ‘high treatment adherence’ group consisted of patients that used the ventilator for more than five hours per night on average and at least 80% of the days in the last three months. Patients that used the ventilator less than five hours per night or less than 80% of the days were grouped with the patients who discontinued HMV in the ‘low treatment adherence’ group. A minimum treatment adherence of five hours per night was chosen because this is associated with better survival in DM1 [5]. Differences between the high treatment adherence group and the low treatment adherence group were analyzed using Pearson’s chi-squared (*χ*^2^) test or Fisher’s exact test for categorical data. The independent-samples *t*-test or the Mann-Whitney U test was used for continuous variables. Logistic regression was performed to identify potential independent predictors of HMV treatment adherence. Covariates with a *p*-value < 0.25 in the univariable analyses were selected: age, BMI, inspiratory positive airway pressure (IPAP), MoCA, CDS and HADS anxiety. Although the *p*-value was <0.25 for expiratory positive airway pressure (EPAP), this variable was not selected, because IPAP was preferred as a representative ventilator setting. Results were quantified as odds ratio (OR) with 95% confidence interval (CI). A *p*-value of <0.05 was considered statistically significant.

## RESULTS

### Patients characteristics

**Fig. 1:**
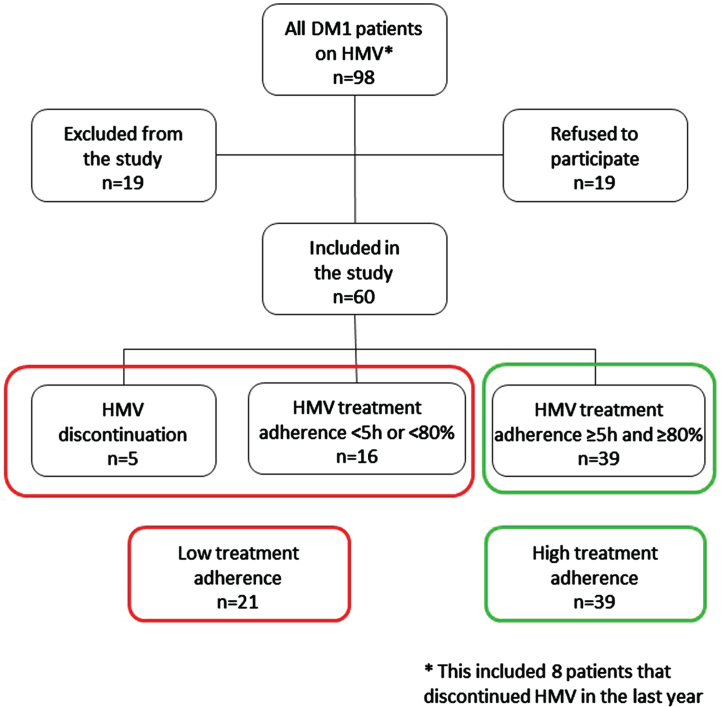
Flow chart of the selection procedure.

Ninety-eight DM1 patients were eligible for participation, including eight patients who discontinued HMV in the past year. Main reasons for discontinuation of HMV were ‘unable to sleep with HMV’ and ‘lack of improvement of symptoms’. Of the 19 excluded patients, six patients were unable to give informed consent, five patients were unreachable, two patients died before participation took place, two patients were medically unstable, two patients were under 18 years of age, one patient participated in another study, and one patient was physically not able to fill in the MoCA. Another 19 patients refused participation. A total of 60 patients was included in the study and subsequently divided into the two predefined groups based on treatment adherence with HMV (Fig. 1). Baseline characteristics and ventilator settings of the cohort are shown in Table 2. Patients with high treatment adherence were on average older (*p* = 0.007) and had a higher BMI (*p* = 0.012) compared to the low treatment adherence group. Out of the 60 included patients, 55 (92%) lived at home, one patient (1%) lived in a nursing home, and four patients (7%) lived in an assisted-living project. With regard to the living situation, there was no difference between the high and low treatment adherence group (*p* = 1.000). Of the patients who lived at home, 11 patients (20%) received professional home care, 16 patients (29%) received care from an informal caregiver and 28 patients (51%) did not have any form of care. With regard to the care situation, there was no difference between the high and low treatment adherence group (*p* = 0.515). Of the total group, half of the patients had a low education level; this did not significantly differ between the low treatment adherence group and the high treatment adherence group (*p* = 0.417). In the high treatment adherence group, the median percentage of days that participants used the HMV was 100 [100–100] and the median number of hours per day was 8.5 [7.3–10]. In the low treatment group, the median percentage of days that participants used the HMV was 51 [0–82], and the median number of hours per day was 3.5 [3.5–5.0].

**Table 2:** Baseline characteristics and ventilator settings

	Total group (*n* = 60)	High treatment adherence (*n* = 39)	Low treatment adherence (*n* = 21)	*p*-value
Age (yrs)^*^	52±12.8	55±11.8	46±12.8	**0.007**
Male	28 (47%)	17 (44%)	11 (52%)	0.515
BMI^*^	29±6.6	31±6.4	26±5.9	**0.012**
CTG repeat length	150 [100–200]	163 [100–200]	150 [100–200]	0.742
50–150	*n* = 15	*n* = 9	*n* = 6	
150–250	*n* = 19	*n* = 12	*n* = 7	
≥250	*n* = 7	*n* = 5	*n* = 2	
DM1 type				
juvenile	*n* = 7	*n* = 4	*n* = 3	
adult-onset	*n* = 50	*n* = 33	*n* = 17	
late-onset	*n* = 3	*n* = 2	*n* = 1	
High MIRS score (4–5)	20 (33%)	14 (36%)	6 (29%)	0.566
pCO_2_ before HMV (kPa)	6.2±0.7	6.1±0.7	6.3±0.6	0.343
FVC sitting (% of predicted)	62±21	61±21	64±20	0.542
FVC supine (% of predicted)	54±21	53±20	56±22	0.596
AHI (nr/h)	19 [10–29]	20 [12–29]	17 [5–31]	0.357
current pCO_2_ (kPa)	5.7±0.8	5.6±0.7	5.7±1.0	0.749
IPAP (cm H_2_O)	17 [14–19]	18 [15–20]	17 [14–18]	0.131
EPAP (cm H_2_O)^*^	7 [6–9]	8 [6–9]	6 [5–8]	**0.026**
Backup frequency	13 [12–14]	13 [12–14]	13 [12–14]	0.953

BMI = body mass index, CTG = cytosine-thymine-guanine, MIRS = muscular impairment rating scale, FVC = forced vital capacity, pCO2 = partial pressure of CO2, AHI = apnea hypopnea index, IPAP = inspiratory positive airway pressure, EPAP = expiratory positive airway pressure; data expressed as mean±SD, frequency (%), or median [IQR]; ^*^p < 0.05.

### Cognitive impairment, affective symptoms, and apathy

**Fig. 2:**
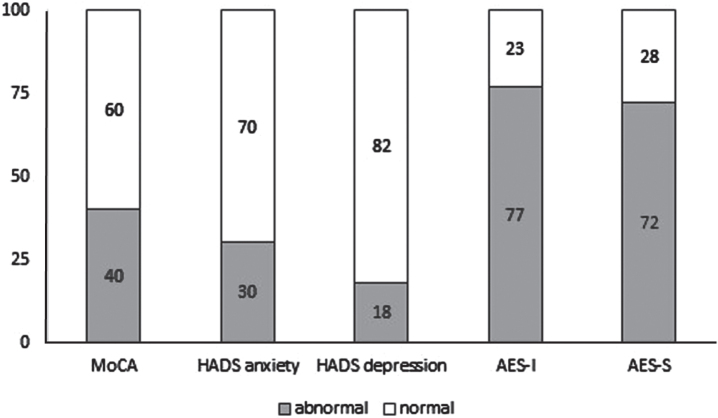
Display of abnormal and normal scores of the main test results. MoCA = Montreal Cognitive Assessment, HADS = Hospital Anxiety and Depression Scale, AES-I = Apathy Evaluation Scale-Informant, AES-S = Apathy Evaluation Scale-Self.

Abnormal scores were found in 40% of the patients for the MoCA and in 72% –77% for the AES, whereas a minority of patients showed abnormal scores for HADS anxiety and HADS depression (Fig. 2). There was no difference between the high treatment adherence group and the low treatment adherence group with regard to the median MoCA score (Table 3). Apathy and depression scores were similar in the high and low treatment adherence group, but the anxiety score was higher in the low treatment adherence group (*p* = 0.012, Table 3). HMV-related side effects, symptoms and sleep quality were equally present in both groups (S^3^-NIV, Table 3). Logistic regression analysis revealed that a higher age and a higher BMI were associated with a greater chance of high treatment adherence (Table 4).

**Table 3:** Cognition, affective symptoms, apathy, HMV related symptoms and side effects

	Total group (*n* = 60)	High treatment adherence (*n* = 39)	Low treatment adherence (*n* = 21)	*p*-value
MoCA	26 [23–28]	26 [23–27]	27 [25–28]	0.148
AES-I	19±6.2	18.4±6.3	18.7±6.2	0.864
AES-S	17±5.0	17.3±5.1	16.3±4.9	0.476
HADS anxiety^*^	4 [2–7]	3 [2–7]	6 [3–8]	**0.012**
HADS depression	4 [2–5]	4 [2.8–6]	4 [2–5]	0.743
CDS	70 [61–73]	69 [60–73]	72 [63–75]	0.107
S^3^-NIV	6.4±1.4	6.5±1.6	6.3±1.1	0.662

MoCA = Montreal Cognitive Assessment, AES-I = Apathy Evaluation Scale-Informant, AES-S = Apathy Evaluation Scale-Self, HADS = Hospital Anxiety and Depression Scale, CDS = Care Dependency Scale, S^3^-NIV = S^3^ noninvasive ventilation questionnaire; data expressed as mean±SD, or median [IQR]; ^*^p < 0.05.

**Table 4:** Logistic regression analysis for the prediction of high treatment adherence

	OR	95% CI	*P* value
Age^*^	1.084	1.013–1.160	**0.019**
BMI (kg/m^2^)^*^	1.151	1.001–1.324	**0.049**
IPAP	1.047	0.839–1.306	0.684
MoCA	1.022	0.812–1.287	0.853
HADS anxiety	0.830	0.663–1.038	0.102
CDS	1.006	0.947–1.068	0.847

IPAP = inspiratory positive airway pressure, MoCA = Montreal Cognitive Assessment, HADS = Hospital Anxiety and Depression Scale, CDS = Care Dependency Scale; ^*^p < 0.05.

## DISCUSSION

This exploratory study showed that cognitive impairment and apathy are frequently present (40% and 77% respectively) in DM1 patients that use HMV, but they are not associated with treatment adherence. Feelings of anxiety were reported in 30% of the study sample and this was found to be associated with low treatment adherence. In addition, higher age and higher BMI were associated with high treatment adherence with HMV. The presence of cognitive impairment has been suggested as a possible factor influencing low treatment adherence in HMV, but this has not been confirmed, nor did we find this in the present study [33]. In daily clinical practice, presence of cognitive impairment in DM1 patients in need of HMV should therefore not be a reason to withhold HMV, but it should be taken into account to ensure adequate support and guidance. Moreover, in patients with CRF, cognitive impairment is common (up to 62%) and may even be partly due to physiological disturbances as a result of the CRF [16]. In these patients, treatment with HMV often leads to improvement of cognitive functioning, further emphasizing the necessity of treating CRF with HMV [16]. In this study, anxiety was associated with low treatment adherence with HMV. Previous studies have been unable to reveal a clear relationship between anxiety and treatment adherence, although this might also have to do with methodological issues [34]. The cause of the elevated anxiety scores in the present study was unknown, leaving the question unanswered whether this is directly related to the CRF and/or the HMV. But it seems plausible that a patient with anxiety complaints may struggle to comply with the prescribed therapy.

Confronting patients with their low treatment adherence and actively asking about anxiety in this context may result in opportunities to improve treatment adherence by offering patients extra support. Higher age and higher BMI were found to be associated with higher treatment adherence in the current study. It has been reported before that treatment adherence is higher when symptoms of respiratory failure are present with initiation of HMV, whereas ‘absence of improvement of symptoms’ often is reported as reason for low treatment adherence or discontinuation of HMV [7, 9]. Because of the progressive nature of DM1, patients with a higher age are more likely to have (more) symptoms of respiratory failure and therefore experience more improvement of symptoms. On the other hand, it should be taken into account that older patients with DM1 might present with the mild (or late-onset) form of DM1 which is associated with less severe symptoms [35]. However, in this study, the vast majority of the patients suffered from the adult-onset form of DM1 (Table 2). In DM1 patients, overweight is an independent risk factor for reduced lung volumes, which can further aggravate respiratory failure [36]. Previous research found that patients with obesity, hypoventilation and symptoms such as sleepiness and dyspnea improve with HMV, as do measures of health-related quality of life [37]. In DM1 patients with a higher BMI, respiratory failure may be more pronounced and symptoms may originate from obesity hypoventilation and therefore lead to more symptom reduction and better treatment adherence. Unfortunately, data on symptoms were not available in the current study. To our knowledge, this is the first time that cognition, affective symptoms, and apathy were studied in the context of treatment adherence with HMV. It is also the first time that the MoCA was used in DM1 patients. Because of the small sample size and the exploratory nature of the study, it is difficult to draw definitive conclusions from this study, but it can create awareness and reject prejudices. Selection bias may have influenced the results, because severely cognitive impaired patients were excluded for this study as they could not provide informed consent. In general, research in the DM1 group is challenging because of the heterogeneity of the disease.

## CONCLUSION

The current study explored the role of cognition, affective symptoms, and apathy in treatment adherence with HMV in DM1 patients. Presence of anxiety was negatively associated with treatment adherence, but no association was found for cognition or apathy emphasizing that HMV should not be withhold in patients with cognitive impairment or signs of apathy. Future research is needed to confirm these results in a larger study sample and should focus on influencing the factors that are negatively associated with treatment adherence with HMV.

## Data Availability

The data supporting the findings of this study are available on request from the corresponding author.
